# Orbital Frontal Cortex Projections to Secondary Motor Cortex Mediate Exploitation of Learned Rules

**DOI:** 10.1038/s41598-018-29285-x

**Published:** 2018-07-20

**Authors:** Drew C. Schreiner, Christina M. Gremel

**Affiliations:** 1 Department of Psychology, University of California San Diego, La Jolla, California 92093 USA; 2 The Neurosciences Graduate Program, University of California, San Diego, La Jolla, California 92093 USA

## Abstract

Animals face the dilemma between exploiting known opportunities and exploring new ones, a decision-making process supported by cortical circuits. While different types of learning may bias exploration, the circumstances and the degree to which bias occurs is unclear. We used an instrumental lever press task in mice to examine whether learned rules generalize to exploratory situations and the cortical circuits involved. We first trained mice to press one lever for food and subsequently assessed how that learning influenced pressing of a second novel lever. Using outcome devaluation procedures we found that novel lever exploration was not dependent on the food value associated with the trained lever. Further, changes in the temporal uncertainty of when a lever press would produce food did not affect exploration. Instead, accrued experience with the instrumental contingency was strongly predictive of test lever pressing with a positive correlation between experience and trained lever exploitation, but not novel lever exploration. Chemogenetic attenuation of orbital frontal cortex (OFC) projection into secondary motor cortex (M2) biased novel lever exploration, suggesting that experience increases OFC-M2 dependent exploitation of learned associations but leaves exploration constant. Our data suggests exploitation and exploration are parallel decision-making systems that do not necessarily compete.

## Introduction

The concepts of exploration and exploitation have been widely studied with focus on the competition between these two processes^1,2^. However, the classical conception of this dilemma^3^ often neglects the possibility that exploratory decisions might utilize previously learned rules and associations. Many tasks which investigate the explore/exploit dilemma are well learned and induce exploration by altering reward delay^4^, magnitude^5^, or probability^6,7^. What is unclear from these tasks is the degree to which animals use learned rules and environmental models to guide their exploration, and how animals might explore in a novel circumstance.

If animals do not generalize learned rules to novel circumstances, what does control exploratory actions, and how do these actions relate to exploitation? The explore/exploit dilemma is classically characterized as a direct trade-off^1^. You are either exploring or exploiting, and doing one necessarily precludes the other. Tasks like the n-armed bandit have reinforced this view, where the mathematically optimal decision (to maximize reward) is defined as “exploit” while all other choices are “explore”^5^. But such a forced choice is rare in the real world. While actions controlled by exploration and exploitation decision processes cannot occur simultaneously, outside of the lab there are often many choice options available that do not explicitly fall into “exploration” or “exploitation”. This raises the possibility that the decision-making aspects of exploration and exploitation run in parallel and do not necessarily directly compete. Thus, it is unclear both the extent to which exploration utilizes information gleaned from the environment, and if and how exploration and exploitation directly compete.

While a large body of work focuses on the explore/exploit dilemma in relation to contextual and cued information, action control may rely on similar processes. The prefrontal cortex has substantial evidence implicating it in learning and applying rules^8–10^ in mediating the explore/exploit dilemma^1,5,11–14^ and in action control^15^. For example, the anterior cingulate cortex has been strongly implicated in the explore/exploit dilemma^4^, while orbital frontal cortex (OFC) and secondary motor cortex (M2) have been implicated in controlling goal-directed instrumental actions^16,17^. It may be that cortical circuits underlying action control could be differentially recruited during explore and exploit processes. Within this framework, OFC has been shown to be necessary for actions sensitive to changing action value^16,18–20^ and partially observable states^21^. M2 has been shown to support goal-directed actions^17^ and the contingency between actions^22–24^. OFC and M2 regions are reciprocally connected^25^, but not onto overlapping populations (i.e. OFC terminal fields in M2 do not overlap with M2 somata that project to OFC, and vice versa)^26^. Furthermore, structural plasticity of OFC projections into M2 (OFC-M2) correlates with rule learning^27^ – specifically, bouton gain correlates with rule learning and subsequent exploitation, while bouton loss correlates with exploration. This suggests that OFC-M2 projections could contribute to or occlude exploration following rule learning.

We used a self-paced operant instrumental lever press task in mice to determine if exploration utilizes learned rules and the extent to which exploration and exploitation directly compete. In this task^28–30^, mice are trained to press one lever for a food reward. Then during the test session a novel but perceptually similar lever is also inserted into the chamber, and we measure responses on the trained and novel levers. Different schedules of reinforcement can be used to bias either exploitation of the trained lever or exploration of the novel lever^28,29^. Previous studies using this particular task have hypothesized that responding reflects either exploration^28,30^ or action generalization mechanisms^29^, though this has not been tested.

We first probed the ability for outcome value to affect responding on the novel lever, and found no evidence that changes in outcome value affect novel lever exploration. Next, we evaluated if temporal uncertainty would affect exploration, and again found no evidence to suggest that temporal uncertainty affects novel lever exploration. Correlative data revealed that the amount of experience mice had with the learned action-outcome rule correlated with exploitation of the trained lever. Importantly, experience did not correlate – either positively or negatively – with exploration. That is, roughly the same level of exploration occurred irrespective of how much experience mice had with the learned rule, indicating that the decision-making processes that mediate exploration and exploitation may not *directly* compete (i.e., more exploitation does not *necessarily* mean less exploration in a free operant context). This led us to examine OFC-M2 projection neurons which, as mentioned, are involved in rule learning^27^. Inhibition of OFC-M2 projection neurons during training and testing increased exploration and reduced exploitation. Overall our data suggest that mice do not generalize previously learned rules when engaging in novel lever exploration, that exploitation and exploration decision processes may run in parallel, and that the OFC-M2 circuit is a critical node controlling the emergence of exploitative action control.

## Results

### Outcome devaluation does not affect lever generalization

We first examined whether mice generalize sensory-specific food outcome expectancies to the novel lever. We took advantage of two different schedules of reinforcement, with a random ratio (RR) schedule biasing sensitivity to sensory-specific changes in food value and a random interval (RI) schedule biasing relative insensitivity to value changes^31,32^. Previous work has found that RR schedules also bias more exploitation of the trained lever while RI schedules bias increased exploration of the novel lever^28,29^. Hence, if mice are generalizing sensory-specific features of the expected food outcome, then outcome devaluation should produce decreased exploratory pressing of the novel lever under an RR schedule in comparison to a RI schedule.

**Figure 1:**
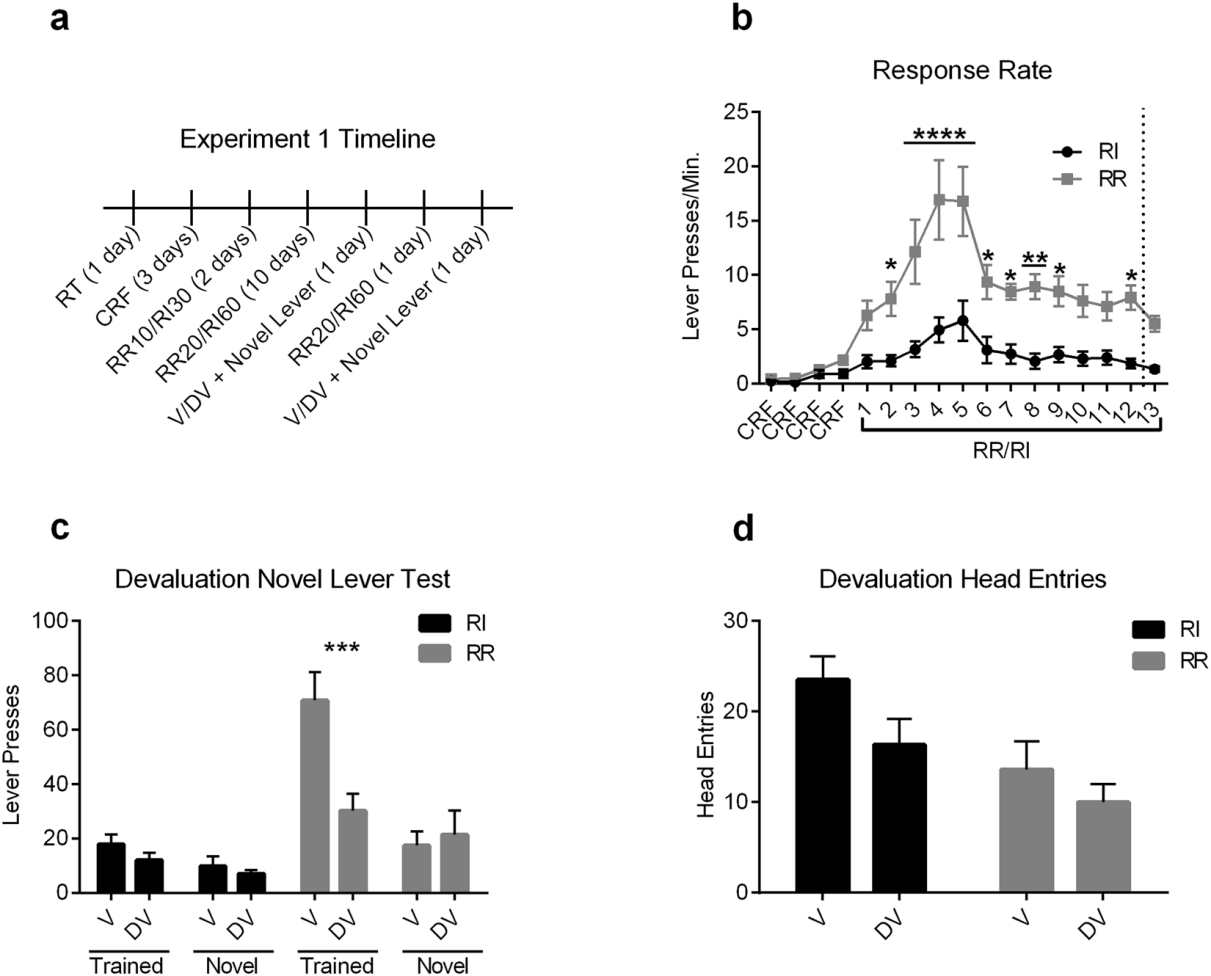
Outcome value does not contribute to novel lever pressing. Mice were trained to press a lever for an outcome under a random ratio (RR) or random interval (RI) schedule and then underwent a combined outcome devaluation/novel lever test. (**a**) Experimental timeline. (**b**) Response rate (Lever Presses/Min.) during acquisition. Days 1–2 were conducted under a RR10/RI30 schedule, remaining days were under a RR20/RI60 schedule. Dotted line indicates where first test day occurred, followed by one day of re-training and then the second test day. Significance markers indicate post-hoc differences between schedules. (**c**) Combined devaluation novel lever test. (**d**) Head entries into the magazine during the combined devaluation novel lever test. RT = Random Time. CRF = Continuous Ratio of Reinforcement. V = Valued Day. DV = Devalued Day. V/DV + Novel Lever = Combined Devaluation Novel Lever Test. Error Bars = ±SEM. n.s. = Not Significant, *p < 0.05, **p < 0.01, ***p < 0.001, ****p < 0.0001.

Mice were trained to press a lever located left or right of a food magazine (counterbalanced) for food pellets under either a RR or RI schedule. Response requirement increased across training, with RI schedules progressing from RI 30 s to RI 60 s, and RR10 progressing to RR20 after two days of schedule training (Fig. 1a). Mice trained under a RR schedule increased their response rate across training to a greater degree than those trained under a RI schedule (Fig. 1b). A two-way repeated-measures ANOVA (Day × Schedule) performed on acquisition response rate (lever presses/minute) revealed a significant interaction (F_(16,224)_ = 5.22, *p* < 0.0001) and significant main effects of Day (F_(16,224)_ = 17.5, *p* < 0.0001) and Schedule (F_(1,14)_ = 19.9, *p* = 0.0005), with post-hoc analyses (Bonferroni corrected) showing schedules differed on most of the training days.

We then performed an outcome devaluation procedure counterbalanced across two days, where the operant outcome is devalued using sensory-specific satiety on the devalued (DV) day, while on the valued (V) day an outcome previously experienced in the homecage is pre-fed to control for effects of general satiation. Following 1 hour free feed access to either the operant or homecage outcome, mice were placed in the operant chamber for a 5 minute extinction test. On both the V and DV day, a second novel lever was inserted (either left or right of the food magazine, counterbalanced) in addition to the trained lever. Mice were re-trained for one day in between the V and DV day.

Outcome devaluation procedures had no effect on exploration of the novel lever in mice trained either on RR or RI schedules (Fig. 1c). A three-way repeated-measures ANOVA (Lever Type × Valuation State × Schedule) showed a significant three-way interaction (F_(1,9)_ = 14.6, *p* = 0.004). A significant two-way interaction between Schedule and Lever Type (F_(1,9)_ = 11.7, *p* = 0.008), showed schedule–induced differences in exploration/exploitation as previously observed^28,29^. There was also a significant interaction between Lever Type and Valuation State (F_(1,9)_ = 19.4, *p* = 0.002), indicating that, overall, only the Trained lever was sensitive to value manipulations. There was no interaction between Schedule and Valuation State (F_(1,9)_ = 3.22, p = 0.11). Main effects of Schedule (F_(1,9)_ = 19.7, *p* = 0.002), Lever type (F_(1,9)_ = 27.7, *p* < 0.001), and Valuation State (F_(1,9)_ = 8.29, *p* = 0.02) were also observed. Planned post-hoc comparisons (Bonferroni corrected) between V and DV days were made for each Lever by Schedule combination. Devaluation significantly reduced Trained lever pressing in RR-trained mice (t_(8)_ = 3.33, *p* = 0.01), but had no effect on Trained lever pressing in RI-trained mice (*p* = 0.23). Devaluation had no effect on Novel lever pressing in either RR (*p* = 0.71) or RI (*p* = 0.52) trained mice.

To determine if a conditioned context-outcome association influenced performance, we also measured head-entries into the magazine. We found no effect of outcome devaluation on the conditioned head-entry response (Fig. 1d). A two-way RM ANOVA (Valuation State × Schedule) showed no significant interaction between Valuation State and Schedule (F_(1,9)_ = 0.303, p = 0.60), nor a significant main effect of Valuation State (F_(1,9)_ = 2.76, p = 0.13), although there was a main effect of Schedule (F_(1,9)_ = 16.3 p = 0.003). Thus, outcome devaluation does not seem to reduce head-entries, suggesting that the context-outcome pairing was not significantly devalued following satiation procedures.

In addition, differences in conditioned response rates acquired between schedules did not contribute to these results (Supplementary Fig. S1). We performed linear regression analyses on average response rate by Devaluation Index (DV index, see methods) to compare the relationship between response rates during training to the degree of outcome devaluation. There was no significant relationship when comparing late acquisition response rate and DV index on the trained (F_(1,9)_ = 2.96, *p* = 0.12; R^2^ = 0.25) or novel (F_(1,9)_ = 0.52, *p* = 0.49; R^2^ = 0.055) lever. Similarly, there was no significant relationship between early response rate and DV index on either the trained or novel lever (Supplementary Fig. S1). Since novel lever presses were lower than trained lever presses, there is the possibility that floor effects could prevent mice from decreasing their novel presses following devaluation. We ran linear regressions of lever press rate during testing on the trained and novel levers with DV Index (for the respective lever). We found no correlation between press rate on the Valued day and DV index for either the trained or novel lever (Supplementary Fig. S1). Likewise, we found no correlation between the average press rate across Valued and Devalued days and DV index for either the trained or novel levers (Supplementary Fig. S1). Hence we found no evidence that response rate during either acquisition or test contributes to the magnitude of outcome devaluation. Outcome devaluation does not appear to affect novel lever exploration, and this was true in mice trained in either a RR or RI schedule, which bias sensitivity or insensitivity (respectively) of trained lever pressing to outcome devaluation.

### Uncertainty does not affect action generalization

Uncertainty is known to modulate the balance between exploration and exploitation^1^. Since previous work has shown that increasing temporal uncertainty (i.e., uncertainty regarding *when* a reward is available) in RI schedules biases the development of habitual actions^33^, and RI schedules promote generalization^28,29^, we hypothesized that increases in temporal uncertainty might lead to increased exploration of the novel lever.

**Figure 2:**
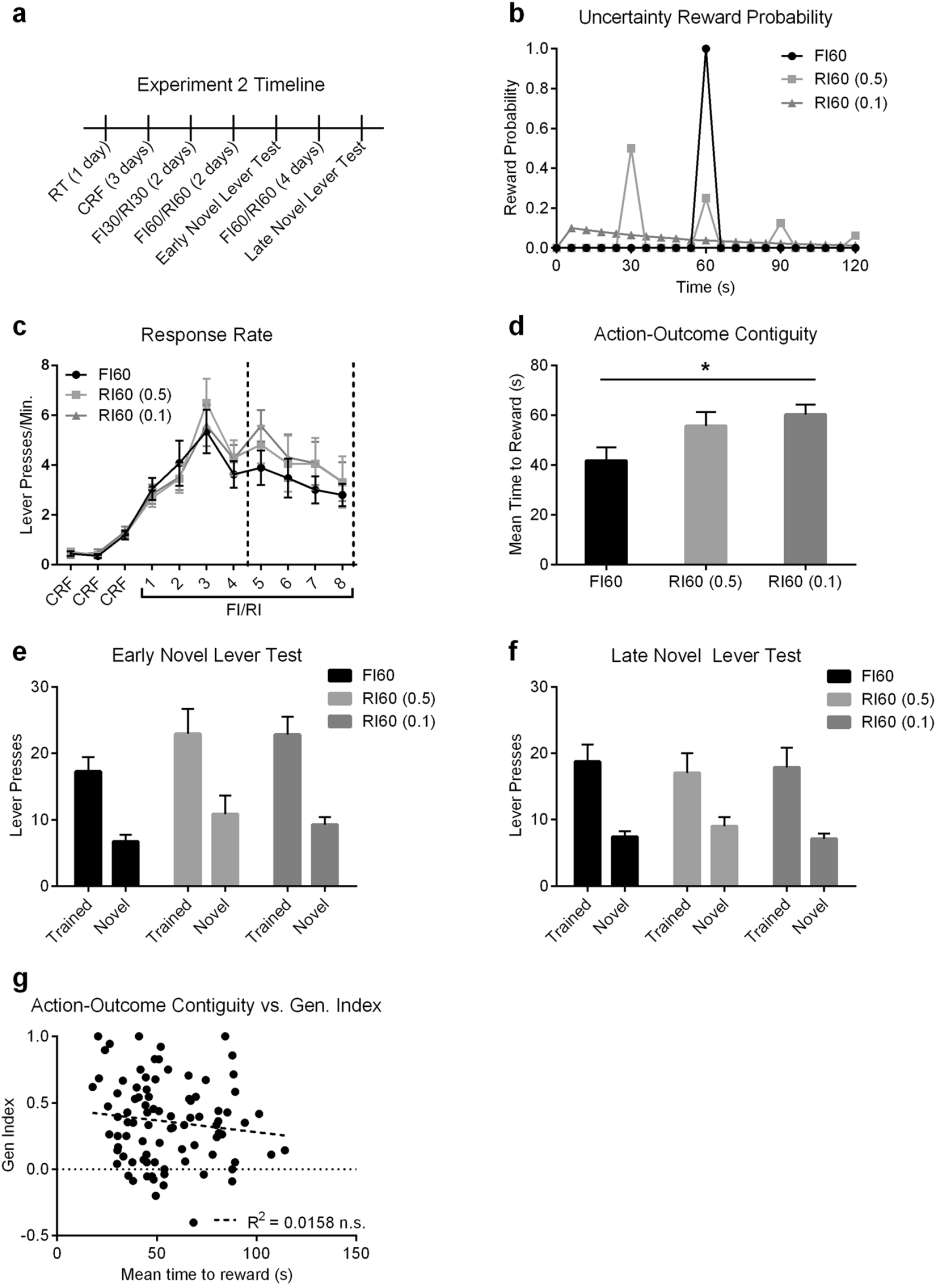
Uncertainty does not contribute to novel lever pressing. Mice were trained to press a lever for an outcome under one of three different interval schedules which varied in their uncertainty. (**a**) Experimental timeline. (**b**) Reward distribution of the three different interval schedules. Note that while the temporal distribution of reward availability differs, all three schedules share the same average time to reward (60 s). (**c**) Response rate during acquisition. Dotted lines indicate where novel lever tests occurred. (**d**) Action-outcome contiguity, defined as mean time between a lever press and reward on the final acquisition day prior to the first novel lever test. (**e**) Early and (**f**) late novel lever test lever presses. In both graphs there is a significant main effect of lever. (**g**) Correlation between action-outcome contiguity and generalization index (Gen. Index), calculated as (Trained Presses − Novel Presses)/Total Presses. FI60 is a Fixed Interval 60 s schedule. RI60 *p* = 0.5 is a Random Interval 60 s schedule with moderate uncertainty. RI60 *p* = 0.1 is a Random Interval 60 s schedule with high uncertainty. RT = Random Time. CRF = Continuous Ratio of Reinforcement. Error Bars = ±SEM. n.s. = Not Significant, *p < 0.05.

Mice were trained under three different schedules (Fig. 2a) that differed in terms of their reward probability distribution, but shared the same average time to reward (Fig. 2b). This was achieved by utilizing different time cycles (T) coupled with different probabilities (p). In the Fixed Interval 60 s schedule (FI60), T = 60 s and *p* = 1.0, such that at every 60 s cycle, there is 100% chance of a reinforcer being earned following a lever press. In the Random Interval 60 s (*p* = 0.5) schedule, T = 30 s and *p* = 0.5, such that at every 30 s cycle, there is a 50% chance of a press producing a reinforcer. In the Random Interval 60 s (*p* = 0.1) schedule, T = 6 s and *p* = 0.1, such that at every 6 s cycle, there is a 10% chance of a press producing a reinforcer. Importantly, the average time to reward is 60 s in all three schedules (Fig. 2b). These schedules did not produce different response rates during acquisition (Fig. 2c), as evidenced by a two-way repeated measures ANOVA (Day × Schedule) that showed no interaction (F_(20,420)_ = 0.64, *p* = 0.89) or main effect of Schedule (F_(2,42)_ = 0.25, *p* = 0.78), but did show a main effect of Day (F_(10,420)_ = 38.7, *p* < 0.0001). We confirmed that our manipulation led to changes in action-outcome contiguity (the average time between a lever press and an outcome delivery)^33^ on the last acquisition day prior to the first novel lever test (one-way ANOVA; significant effect of schedule (F_(2,41)_ = 3.86, *p* = 0.029) (Fig. 2d). Hence mice learned to press the lever under different degrees of temporal uncertainty.

We found no evidence to suggest that temporal uncertainty affects exploration of the novel lever. Mice were given two novel lever tests where an additional, novel lever was inserted into the chamber along with the trained lever; an early test was conducted after initial acquisition at a time point early on in rule learning, and a second late test was conducted after extended training, although in this case the additional lever was not completely novel. A two-way repeated measures ANOVA (Lever Type × Schedule) conducted on lever presses in the early test did not show an interaction (*p* = 0.77) or a main effect of Schedule (*p* = 0.16), but did show a main effect of Lever Type (F_(1,42)_ = 47.7, *p* < 0.0001) (Fig. 2e). Similarly, a two-way repeated measures ANOVA conducted on lever pressing during the late test did not show an interaction (*p* = 0.73) or main effect of Schedule (*p* = 0.96), but did show a significant main effect of Lever Type (F_(1,42)_ = 33.5, *p* < 0.0001) (Fig. 2f). As these three interval schedules have been demonstrated to differ in their action-outcome contiguity^33^ (Fig. 2d), we correlated action-outcome contiguity with Generalization Index (Gen. Index: values close to 1 indicate complete exploitation of the trained lever, while values near 0 indicate generalized responding to both levers, see methods). We found no correlation between the action-outcome contiguity on the last training day and the degree to which mice generalized lever pressing to the novel lever during testing (F_(1,88)_ = 1.40, *p* = 0.24; R^2^ = 0.02) (Fig. 2g). Overall, our data show mice exhibited weak generalization of responding, and we found no evidence that temporal uncertainty influenced novel lever exploration.

### Action Experience Biases Selective Exploitation

**Figure 3:**
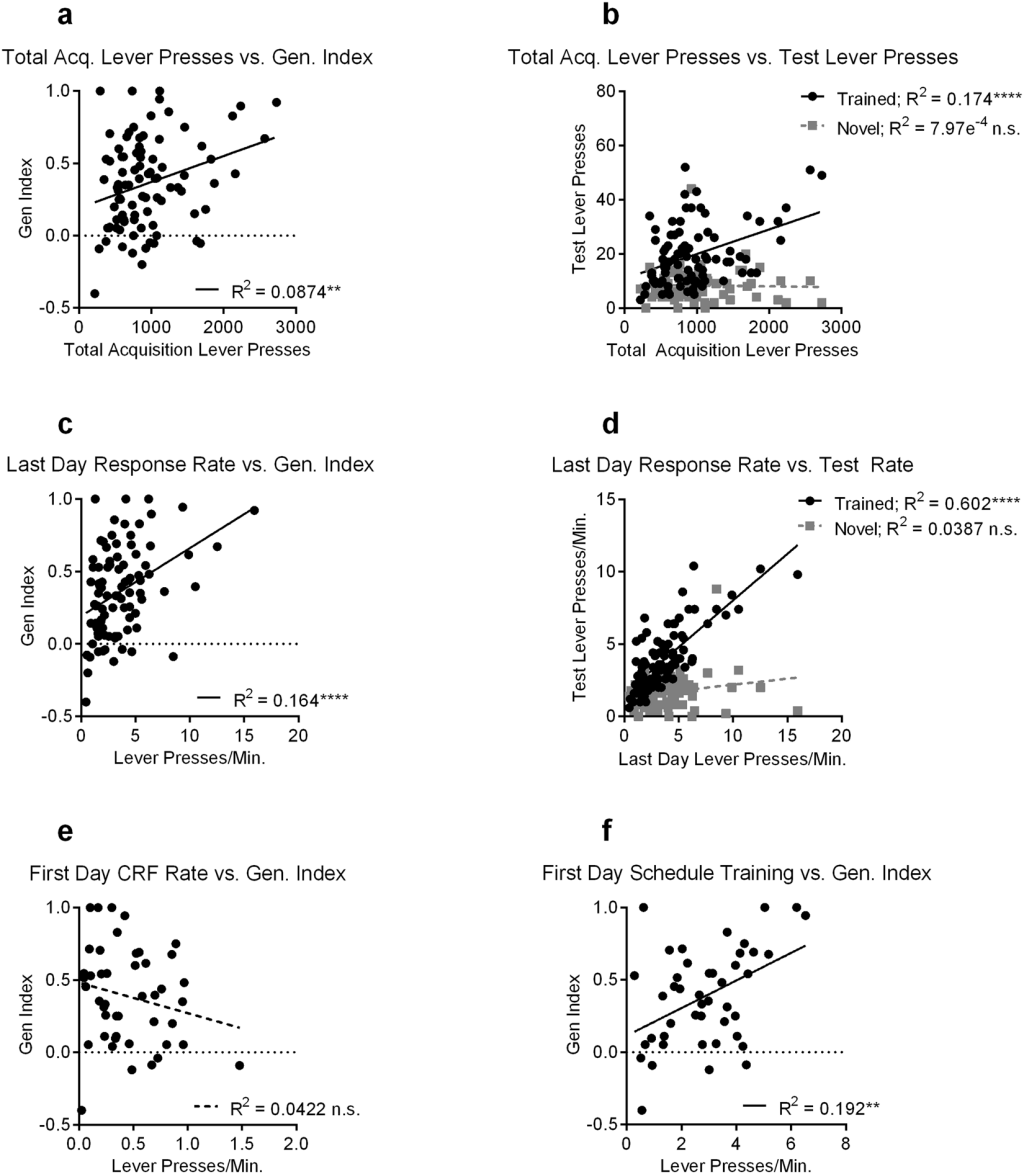
Experience with the trained lever correlates with exploitation but not exploration. The same mice from Fig. 2 were used to run these correlations. (**a**) Correlation between total lever presses during acquisition and generalization index (Gen. Index). (**b**) Correlation between total lever presses during acquisition and test lever presses on the trained or novel lever. (**c**) Correlation between last day response rate and generalization index. (**d**) Correlation between last day response rate and test response rate on trained and novel levers. (**e**) Correlation between response rate on the final CRF (Continuous Ratio of Reinforcement) training day and generalization index. (**f**) Correlation between response rate on the first day of schedule training and generalization index. Dotted linear regression lines indicate non-significant correlations, while solid linear regression lines are significant. Acq. = Acquisition. n.s. = Not Significant, **p < 0.01, ****p < 0.0001.

We next sought to determine if the amount of experience with the learned action biased towards exploitation, as previously reported^30^. Utilizing data obtained from the mice in the uncertainty experiment above, we calculated the total lever presses made since the start of schedule training until either the early or late generalization test. We found that experience with the learned action did indeed bias towards exploitation. A linear regression analysis of total lever presses during acquisition and the generalization index revealed a small but significant positive relationship (F_(1,88)_ = 8.43, *p* = 0.005; R^2^ = 0.087) (Fig. 3a), with more total lever presses during acquisition leading to higher generalization index values (i.e., more exploitation). We ran separate linear regressions broken up by training schedule (FI vs. RI (0.5) vs. RI (0.1)) to determine if this effect was primarily driven by one schedule. We found that there was still a significant relationship between total lever presses during acquisition and generalization index in the FI (F_(1,28)_ = 6.67, *p* = 0.015; R^2^ = 0.19), and the RI(0.1) (F_(1,32)_ = 5.88, *p* = 0.02; R^2^ = 0.16) schedules, but not in the RI(0.5) schedule (F_(1,26)_ = 0.03, *p* = 0.86; R^2^ = 0.0013) (Supplementary Fig. S2). This demonstrates that this relationship is not driven by only one schedule, and indeed is observed in the schedules that differ most in terms of their uncertainty (that is, uncertainty does not appear to contribute to the correlation between experience and exploitation).

An increased generalization index could indicate either an increase in trained lever presses and/or a decrease in novel lever presses. We therefore ran linear regressions using total lever presses during acquisition by trained or novel lever presses collapsed across early and late tests (Fig. 3b). Interestingly, we found a significant relationship with only trained lever presses (F_(1,88)_ = 18.5, *p* < 0.0001; R^2^ = 0.17), and not with novel lever presses (F_(1,88)_ = 0.07, *p* = 0.79; R^2^ = 7.97e-4). Furthermore, the slope of these two lines (trained vs. novel lever press) differed significantly (F_(1,176)_ = 15.1, *p* = 0.0001), indicating that the amount of experience with the trained lever is highly predictive of trained lever presses on test, but does not impact the degree of novel lever exploration. Indeed, this relationship was present on the last day of training prior to testing, where we again find a significant relationship between last day response rate and generalization index (F_(1,88)_ = 17.2, *p* < 0.0001; R^2^ = 0.16) (Fig. 3c), and with test response rates on the trained (F_(1,88)_ = 133, *p* < 0.0001; R^2^ = 0.60) but not the novel (F_(1,88)_ = 3.54, *p* = 0.06; R^2^ = 0.04) lever, and again the slopes of these two lines differed significantly (F_(1,176)_ = 62.5, *p* < 0.0001) (Fig. 3d).

We next sought to determine how early this relationship between response rate and generalization index emerged. For these analyses, we used data only from the early generalization test to examine the relationship between initial learning and testing. Using response rates from the very first day of CRF (Continuous Ratio of Reinforcement) training, we found no significant relationship with the subsequent generalization index (*p* = 0.18, R^2^ = 0.04) (Fig. 3e). This lack of a significant relationship persisted throughout the following 2 days of CRF training (Supplementary Fig. S2), though it should be noted that the low response rates during this initial CRF training might make correlations difficult to detect. However, by the first day of schedule training on FI30 or RI30, a significant relationship between response rate and the generalization index emerged (F_(1,43)_ = 10.2, *p* = 0.003; R^2^ = 0.19) (Fig. 3f). This suggests that differences in the action-outcome relationships experienced during early schedule learning contribute to exploitation on the trained lever.

These results indicate that the amount of experience with a known action-outcome relationship is predictive of the subsequent degree of exploitation during a probe test, with more experience and higher rates of responding correlating with increased exploitation of the trained lever. However, there was no correlation with exploration, as might be expected if actions were being generalized. Similarly, if exploitation and exploration decision-processes directly competed with one another, we should expect to see a negative correlation (that is, as exploitation increases with experiences, exploration should decrease), but instead we see no relationship between experience and exploration whatsoever. When we measured the duration mice hold the trained versus novel lever down in a separate cohort of mice, we found that lever press durations can differ between trained and novel levers (Supplementary Fig. S3), indicating that the motor response itself may not fully generalize. Together, the results of our uncertainty experiment provide evidence that the learned Stimulus-Response association does not generalize to the novel lever

### Orbital frontal cortex projections to secondary motor cortex mediate learned action-outcome associations

Our data suggests that increased experience drives exploitation of known rules. Rule learning in uncertain environments has been proposed to induce structural plasticity of OFC terminals in M2, with the magnitude of this plasticity correlating with subsequent exploitation of known rules^27^. We hypothesized that activity of OFC projections to M2 is necessary for rule learning that supports exploitation of the trained lever. Hence, inhibiting OFC projections to M2 during both learning and testing should occlude this plasticity, and thereby bias exploration during the novel lever test.

**Figure 4:**
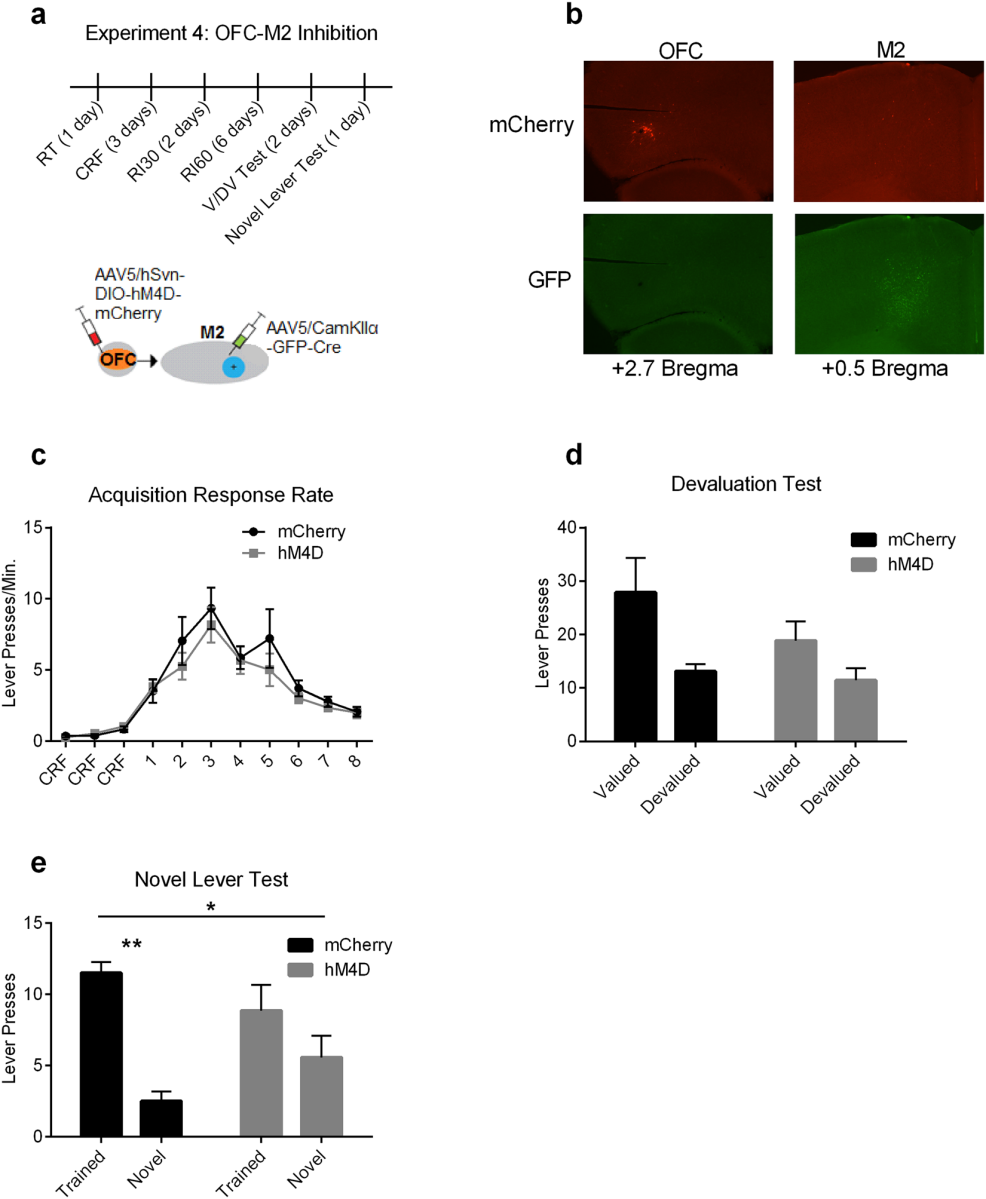
Chemogenetic attenuation of OFC-M2 projection neurons reduces exploitation of learned rules. (**a**) (top) Experimental timeline and (bottom) schematic of dual viral vector injection. (**b**) Representative images of mCherry and GFP fluorescence at 3.2x magnification in both OFC and M2. (**c**) Response rate during acquisition. mCherry = Fluorophore control mice expressing mCherry. hM4D = Inhibitory DREADD-expressing mice. (**d**) Lever presses during outcome devaluation. There is a significant main effect of Valuation State. (**e**) Lever presses during novel lever test. RT = Random Time. CRF = Continuous Ratio of Reinforcement. RI = Random Interval. V/DV = Outcome Devaluation Test. Bars = ±SEM. n.s. = Not Significant, *p < 0.05, **p < 0.01.

We utilized a dual viral vector approach to isolate OFC projections into M2, and used chemogenetics to specifically attenuate OFC-M2 activity (Fig. 4a). Mice were given bilateral injections in OFC of a rAAV5/hSyn-DIO-hM4D-mcherry expressing a Cre-dependent inhibitory Designer Receptor Exclusively Activated by a Designer Drug (DREADD)^34^ or a rAAV5/hSyn-DIO-mcherry expressing a Cre-dependent fluorophore control (mCherry). In M2, all mice received bilateral injections of AAV5/CamKIIα-GFP-Cre expressing GFP-Cre under the control of the CamKIIα promoter that can be transferred retrograde^35^. We observed minimal expression of neurons which project in the other direction (M2 to OFC: as evidenced by lack of mCherry in M2 and lack of GFP in OFC; Fig. 4b).

All mice were trained under a RI schedule. All animals received injections of the hM4D agonist CNO (1.0 mg/ml) 30 minutes prior to all schedule training and test days, a duration we have previously shown sufficient to reduce OFC cell excitability^16,18^. hM4D and mCherry control mice showed similar acquisition of lever press behavior (Fig. 4c). A two-way repeated measures ANOVA (Day × Virus) did not show a significant interaction (*p* = 0.82), or main effect of Virus (*p* = 0.46), but did show a main effect of Day (F_(10,130)_ = 25.3, *p* < 0.0001). Since both OFC^16^ and M2^17^ are individually necessary for goal-directed actions under outcome devaluation, we first sought to test if the projections from OFC to M2 were specifically necessary for goal-directed actions. We took advantage of previous findings that action control relatively early in training under RI schedules is still goal-directed^36,37^, and performed outcome devaluation procedures after relatively little training. A two-way repeated measures ANOVA (Valuation state × Virus treatment) showed no interaction (*p* = 0.31), nor a main effect of virus (*p* = 0.26). Only a main effect of Valuation State (F_(1,13)_ = 10.1, *p* = 0.007) was observed, indicating that OFC-M2 activity attenuation during training and testing did not disrupt goal-directed control (Fig. 4d).

Following devaluation testing, we next assessed the involvement of the OFC-M2 projection in a second test session in which the novel lever was introduced. In contrast to our outcome devaluation results, we found that attenuation of OFC-M2 projection neuron activity decreased exploitation of the trained lever in relation to exploration of the novel lever (Fig. 4e). While mCherry control mice pressed the trained lever to a much greater degree than the novel lever, hM4D mice pressed each of the levers a similar amount of times. A two-way repeated measures ANOVA (Lever × Virus), revealed a significant interaction (F_(1,13)_ = 5.97, *p* = 0.03) and significant main effect of Lever (F_(1,13)_ = 27.6, *p* = 0.0002), but no main effect of Virus (*p* = 0.87). Bonferroni-corrected post-hoc testing revealed that only mCherry control mice differentially distributed their presses between the trained and the novel lever (t_(13)_ = 5.63, adjusted *p* = 0.0002), while the hM4D mice did not (adjusted *p* = 0.15). These results indicate that the OFC-M2 projection is functionally involved in learning to exploit known rules in an uncertain environment.

## Discussion

Our data suggests that exploitation and exploration are parallel decision processes, with OFC-M2 circuits supporting the acquisition and performance of exploitation. We have provided multiple, convergent lines of evidence indicating that mice do not generalize learned action contingencies in total during exploration on a novel lever, but instead choose to exploit known rules while they continue to explore for new rules associated with a novel lever. In support of this, learned outcome value of the trained lever does not appear to control novel lever pressing, nor does the amount of uncertainty experienced during learning. Instead, we find that experience with the learned rule predicts subsequent exploitation of that lever during testing, while that experience has little effect on continued exploration. In agreement with this, chemogenetic inhibition of OFC neurons projecting to M2 – a neural circuit involved in rule learning – was sufficient to induce greater exploration.

Attenuation of the OFC-M2 circuit revealed a functional role for this circuit in biasing exploitation of known rules. To our knowledge, this is the first time this circuit has been functionally manipulated whatsoever. OFC has a long history of research implicating it in representing outcome value^38^ and in reversal learning^39^, and has recently been proposed to incorporate expected uncertainty during decisions to guide behavior^40^. A prominent hypothesis has been that the OFC represents the state space of a given task^41^. With regards to the latter hypothesis, an unanswered question is, where does OFC convey this state space information? OFC projections into amygdala^42^, and dorsal striatum^18^ appear to convey information necessary for value-based decision-making, including broader state space representations^43^. Intracortical OFC projections have been largely neglected, but are interesting candidate regions for the conveyance of this state space information. One such cortical region is M2, which has been proposed to utilize evidence – both external sensory and internal information – to guide actions^44^. What is unclear is whether M2 is directly computing and utilizing evidence, or whether this information arrives from other regions^45,46^. OFC is an interesting candidate source, given that M2 and OFC are reciprocally connected^25^, and bouton gain of OFC axons in M2 positively correlates with exploitation of learned rules, while bouton loss correlates with exploration^27^. This provides correlative evidence that OFC is indeed conveying task-relevant information to M2. Our results provide a causal link between activity in this pathway and subsequent decision-making, suggesting contribution of the OFC-M2 projection in arbitrating the exploitation of learned rules. Since we inhibited OFC-M2 projections throughout both training and test, we cannot conclude if this projection is also involved in using this learned information during novel lever testing. However, the results of the structural plasticity study^27^ would indicate that OFC-M2 projections are specifically involved in learning, particularly since there was no differences in structural plasticity between groups of mice that had to recall an already known rule vs. those that underwent a reversal.

We found no evidence for the involvement of OFC-M2 projections in goal-directed decision-making following outcome devaluation. This is somewhat surprising, as both OFC^16^ and M2^17^ are individually necessary for goal-directed control following outcome devaluation. In agreement with our current results, structural plasticity of OFC projection neurons in M2 does not correlate with the experience of reward alone, but instead specifically correlated with learning the relationship between actions and outcomes^27^. Thus it appears that while OFC projections into dorsal striatum^18^ and amygdala^42^ are involved in using value change to guide actions, we find no evidence that OFC projections to M2 convey outcome value; instead they may encode learned rules among outcomes, actions, and stimuli. Therefore, our findings suggest a projection-specific dissociation of OFC function, as we identify an OFC projection which may utilize state space representations provided by OFC to guide decision-making and action selection.

The results of our combined novel lever test and outcome devaluation study find no evidence that outcome value influences novel lever exploration. These results are significant on several different levels. Firstly, they replicate the finding that RR schedules bias goal-directed control over behavior and selective exploitation of the trained lever during a novel lever test, while RI schedules bias habitual control over behavior and exploration of the novel lever^28,29,31^. Thus, we were able to combine the novel lever test with the devaluation test and still replicate classical and long-standing schedule-induced differences in action control. This combination could prove useful, as it allows for the simultaneous study of different action control systems. This experiment also indicates that learned action-outcome associations do not generalize to the novel lever, as outcome value manipulations – which control responding on the trained lever – have no effect on novel lever exploration.

It has been proposed that generalization on the novel lever test might occur as a result of a learned stimulus-response association generalizing to the perceptually similar novel lever^29^. However, we find that temporal uncertainty, which is known to increase habitual control over behavior^33^ has no effect on novel lever pressing. Additionally, we measured the duration of lever presses themselves (i.e., the response) in a separate experiment, and discovered that mice trained in RR schedules press the trained and novel lever differently. Thus, performance of the learned response itself does not completely generalize to the novel lever. It seems therefore that neither the stimulus-response relationship nor the response itself are fully generalized to the novel lever.

If novel lever pressing is not the sole result of generalization of learned rules, or of stimulus-response associations, what *is* controlling responding? It has recently been proposed that exploration is a distinct, early stage of learning which disappears following extended training^30^. If exploration disappeared with training, we should expect a negative correlation between the amount of experience an animal had with the instrumental contingency and novel lever pressing. While we find evidence that the amount of experience correlates with trained lever pressing, there is no such relationship with novel lever pressing. Put another way, roughly the same level of novel lever exploration occurs regardless of the amount of experience animals have with the trained lever. Thus, animals might appear to explore early in training simply because there is relatively less exploitation occurring at this time point.

Classically, the explore/exploit dilemma is treated as a zero-sum game, where one necessarily excludes the other. While animals of course cannot simultaneously make explore/exploit-related actions, the trade-off between the two is not strictly zero sum as evidenced in the self-paced operant task used in this study. Mice in our task (and animals foraging in the wild) have many potential actions available to them – grooming, locomotion, making head entries – that do not explicitly fall into exploitation or exploration. It could be that the trial-based, forced choice structure of many tasks forces the apparent direct trade-off between exploration and exploitation. Our results suggest that the decision-making processes that arbitrate exploration and exploitation may not inherently be in competition; rather, they may run in parallel with action selection arising from the winning decision made^47^. This is analogous to the current understanding of goal-directed and habitual action control systems as parallel processes, either of which may contribute to action control at a given time point^15^. If exploration and exploitation decision processes do indeed run in parallel, an intriguing prediction is that it should be possible to selectively manipulate one or the other of these processes.

In support of this view, many studies have found different neuroanatomical substrates for exploration and exploitation^2,5,12,13^. However, other regions like the locus coeruleus (LC) have been implicated in both exploration and exploitation^48^. Interestingly, the LC is reciprocally connected with OFC^48^ and M2^49^. It has been proposed that cortical input into LC is crucial for its ability to shift behavior between exploration and exploitation^48^, and LC input into anterior cingulate (and adjacent M2) is critically involved in increasing behavioral variability that could underlie exploration^50^. LC norepinephrine is an important modulator of plasticity in the brain^51^; it is unknown if OFC-M2 projection plasticity might also be sculpted by LC norepinephrine input during learning.

We have provided evidence that novel exploration is unlikely to fully utilize previously learned rules about actions from the environment. This raises the possibility that the decision-making processes that arbitrate between exploration and exploitation may run in parallel and may not directly compete with one another.

## Methods

### Animals

Similar numbers of male and female C57BL/6J mice (>7 weeks/50 PND) (The Jackson Laboratory, Bar Harbour, ME) were used for experiments. All procedures were conducted during the light period and mice had free access to water throughout the experiment. Mice were food restricted to 90% of their baseline weight 2 days prior to the start of experimental procedures, and were fed 1–4 hours after the daily training. All experiments were approved by the University of California San Diego Institutional Animal Care and Use Committee and were carried out in accordance with the National Institutes of Health (NIH) “Principles of Laboratory Care”. Mice were housed 2–4 per cage on a 14:10 light:dark cycle.

### Acquisition

Mice were trained once per day in operant chambers in sound attenuating boxes (Med-Associates, St Albans, VT) in which they pressed a lever (left or right of the food magazine, counterbalanced for location) for an outcome of regular ‘chow’ pellets (20 mg pellet per reinforcer, Bio-Serv formula F0071). Each training session commenced with an illumination of the house light and lever extension and ended following schedule completion (30 reinforcers) or after 60–90 minutes had elapsed with the lever retracting and the house light turning off.

On the first day, mice were trained to approach the food magazine (no lever present) on a random time (RT) schedule, with a reinforcer delivered on average every 60 seconds for a total of 30 minutes. Next, mice were trained on a continuous ratio schedule of reinforcement (CRF) across 3 days, where every lever press was reinforced, with the total possible number of earned reinforcers increasing across days (CRF 5, 15, and 30).

Following CRF, mice were trained on either a random interval (RI) schedule to bias habitual control over actions^32^ and action generalization^29^, or a random ratio schedule (RR) to bias goal-directed action control and action exploitation. In a RI(Y) schedule, the first lever press after an average of (Y) time has elapsed will be reinforced, using a probability distribution of *p* = 0.10 (e.g. in RI30, the first lever press after 30 seconds – on average – have elapsed will be rewarded). In a RR(X) schedule, on average (X) lever presses must occur before a reward is delivered. Initial training was conducted on a RI30 and RR10 for two days, followed by a progression to RI60 and RR20 (see each experiment for timeline details).

### Generalization Testing

As described previously^29^, mice were placed in the training context and at session start two levers were extended; the previously trained lever as well as a novel, but identical lever in a different spatial location. Testing took place over 5 minutes and was conducted in the absence of reinforcement. Mice that made 0 presses on the trained lever were excluded from analyses.

### Outcome Devaluation

Devaluation procedures occurred across two days. In brief, on the valued day, mice had ad libitum access to an outcome previously experienced in the home cage for 1 hour before being placed in the training context for a 5 minute, non-reinforced test session. On the devalued day, mice were given 1 hour of ad libitum access to the outcome previously earned by lever press, and then underwent a 5 minute, non-reinforced test session in the training context. The order of revaluation day was counterbalanced across mice. Mice who did not consume at least 0.1 g of food on either the valued or devalued day were excluded.

### Combined Outcome Devaluation and Generalization

Outcome devaluation was combined with the novel lever test such that both the trained and novel lever were presented following outcome devaluation via specific satiety. Testing occurred across two days, separated by one day of re-training in between. All conditions were counterbalanced between days.

### Drugs

The hM4D-selective agonist Clozapine N-Oxide (CNO) was obtained from the National Institute of Mental Health (Bethesda, MD). The CNO dosage was 1.0 mg/kg at 10 ml/kg per mouse, delivered in saline via intraperitoneal injection. All mice were pretreated with CNO 30 minutes prior to the start of training or testing to allow for CNS penetration^16^.

### Surgical Procedure

For chemogenetic attenuation of OFC-M2, all viral vectors were obtained from the UNC Viral Vector Core (Chapel Hill, NC). Mice were anaesthetized with isoflurane (1–2%) and bilateral intracranial injections were performed via Hamilton (Reno, NV) syringe targeted at M2 (from Bregma: AP +0.5 mm, L ±0.5 mm and V −1.25 mm from the skull), or OFC (from Bregma: AP +2.7 mm, L ±1.65 mm and V −2.65 mm from the skull). Mice (n = 16) received 200 nl of a viral vector (rAAV5/CamKIIα-GFP-Cre) expressing Cre recombinase (Cre) under the control of the calcium calmodulin dependent protein kinase II α (CamKIIα) in M2. In OFC, n = 8 mice received 200 nl of a viral vector (rAAV5/hSyn-DIO-mcherry) as a control, and n = 8 mice received 200 nl of a viral vector (rAAV5/hSyn-DIO-hM4D-mcherry) expressing a Cre-inducible, inhibitory DREADD (hM4D) coupled to a G_i_ signaling cascade which induces neuronal attenuation^34^. Syringes were left in place for five minutes after injection to allow for diffusion. Mice were given at least two weeks of recovery before the start of experimental procedures. After behavioral testing was concluded, mice were euthanized and brains were extracted and fixed in 4% paraformaldehyde. The hM4D virus expressed the fluorescent marker mCherry, while the Cre virus expressed the fluorescent marker GFP. Localization and spread of viral expression was assessed in 100 µm thick brain slices using fluorescent microscopy (Olympus MVX10). The final n’s were: n = 7 hM4D mice and n = 8 mCherry control mice.

### Data analysis

For all analyses, α = 0.05 was used as a threshold for significance. All analyses were two-tailed. Initial analyses were conducted to assess normal distributions and similar standard deviations. Where we found evidence for non-normal distributions or different standard deviations, we used Mann-Whitney tests. One-way or two-way repeated measures ANOVAs were used to examine acquisition and test data unless stated otherwise. The devaluation index was calculated by subtracting lever presses on the devalued day (DV) from lever presses on the valued day (V) and dividing by the total number of lever presses across both days (V − DV)/(V + DV). The generalization index was calculated by subtracting novel lever presses from trained lever presses and dividing by the total number of lever presses (Trained − Novel)/(Trained + Novel). Action-outcome contiguity was calculated by measuring the time in between a lever press and the next reinforcer delivery on average per animal. Behavioral data was recorded by MED-PC IV software, and analyzed in Excel, Matlab (Mathworks), Prism (Graphpad), and JASP.

### Experiment 1: Role of outcome value in action generalization

16 C57BL/6J mice were used for this experiment. Two days prior to the start of behavioral procedures, mice were habituated to a novel cage for 1.5 hours which would later be used in the devaluation procedure. On schedule training days, mice were given a non-contingent, home cage outcome of 20% w/v sucrose (Sigma Aldrich, St. Louis, MO) 1–4 hours after training, which would serve as a control for satiety during the devaluation test. Half of the subjects (n = 8) were trained under a RR schedule, while the other half (n = 8) were trained under a RI schedule of reinforcement. Mice were trained for 2 days on either RR10 or RI30, before being switched to a RR20 or RI60 schedule for 10 days of training prior to the combined outcome devaluation, action generalization test (Fig. 1a). During the devaluation generalization test, several mice were excluded due to failing to consume the minimum during pre-feeding (0.1 g either day), giving a final sample size n = 6 RI and n = 5 RR during the test.

### Experiment 2: Role of uncertainty in action generalization

48 C57BL/6J mice were used for this experiment. Subjects were broken up into three different uncertainty groups using interval schedules of reinforcement, each with an initial n = 16. The three schedules used were a Fixed Interval (FI), a RI *p* = 0.5 and a RI *p* = 0.1 as described previously^33^. 3 mice were excluded for failing to acquire the task (1 from FI, 2 from RI *p* = 0.5) to give final sample sizes of n = 15 FI, n = 14 (RI *p* = 0.5), and n = 16 (RI *p* = 0.1). The schedules differed in terms of their reward probability distribution, but all shared the same average time to reward (Fig. 2b). This was achieved by utilizing different time cycles (T) coupled with different probabilities (p). In the FI60 schedule, T = 60 s and *p* = 1.0, such that at every 60 s cycle, there is 100% chance of a reinforcer being earned following a lever press. In the RI60 (*p* = 0.5) schedule, T = 30 s and *p* = 0.5, such that at every 30 s cycle, there is a 50% chance of a press producing a reinforcer. In the RI60 (*p* = 0.1) schedule, T = 6 s and *p* = 0.1, such that at every 6 s cycle, there is a 10% chance of a press producing a reinforcer. Mice were pre-trained on a RT and CRF schedule as described above, before being switched onto a FI30/RI30 schedule for 2 days, followed by 2 days of a FI60/RI60 schedule, then 1 day of novel lever testing, then 4 additional days of FI60/RI60 training, followed by a final day of novel lever testing (Fig. 2a).

### Experiment 3: Schedule-induced differences in action performance

7 C57BL/6J mice were used for this experiment, with n = 4 trained under a RI schedule and n = 3 trained under a RR schedule. During this experiment, the lever press durations were recorded. Mice were trained for 2 days on a RR10/RI30 schedule, followed by 10 days of RR20/RI60 training, followed by a novel lever test.

### Experiment 4: Role of OFC to M2 projections in action generalization

16 C57BL/6J mice were used for this experiment. One hM4D mouse was excluded due to poor fluorophore expression leaving final n’s at n = 8 mCherry controls and n = 7 hM4D mice. After pre-training, mice were trained for two days on a RI30 schedule, followed by 6 days of training on a RI60 schedule, followed by outcome devaluation testing. The following day, mice underwent a novel lever test (Fig. 4a). CNO pretreatment began on the first day of schedule training and continued throughout training and testing.

### Data availability

The datasets generated and code used during the current study are available from the corresponding author on reasonable request.

## Electronic supplementary material

Supplementary Information
