# Supplementary material for: Orbital Frontal Cortex Projections to Secondary Motor Cortex Mediate Exploitation of Learned Rules
Source: Sci Rep. 2018 Jul 20;8:10979. doi: 10.1038/s41598-018-29285-x (PMC6054681; doi:10.1038/s41598-018-29285-x)
Supplement: Supplementary file 1 — Supplementary Information [file 41598_2018_29285_MOESM1_ESM.pdf]

Orbital Frontal Cortex Projections to Secondary Motor Cortex

Mediate Exploitation of Learned Rules

Drew C. Schreiner[1] and Christina M. Gremel*[1, 2]

[1]Department of Psychology, University of California San Diego, La Jolla, California, 92093

[2]Neurosciences Graduate Program, University of California, San Diego, La Jolla, California, 92093

Supplementary Figure S1

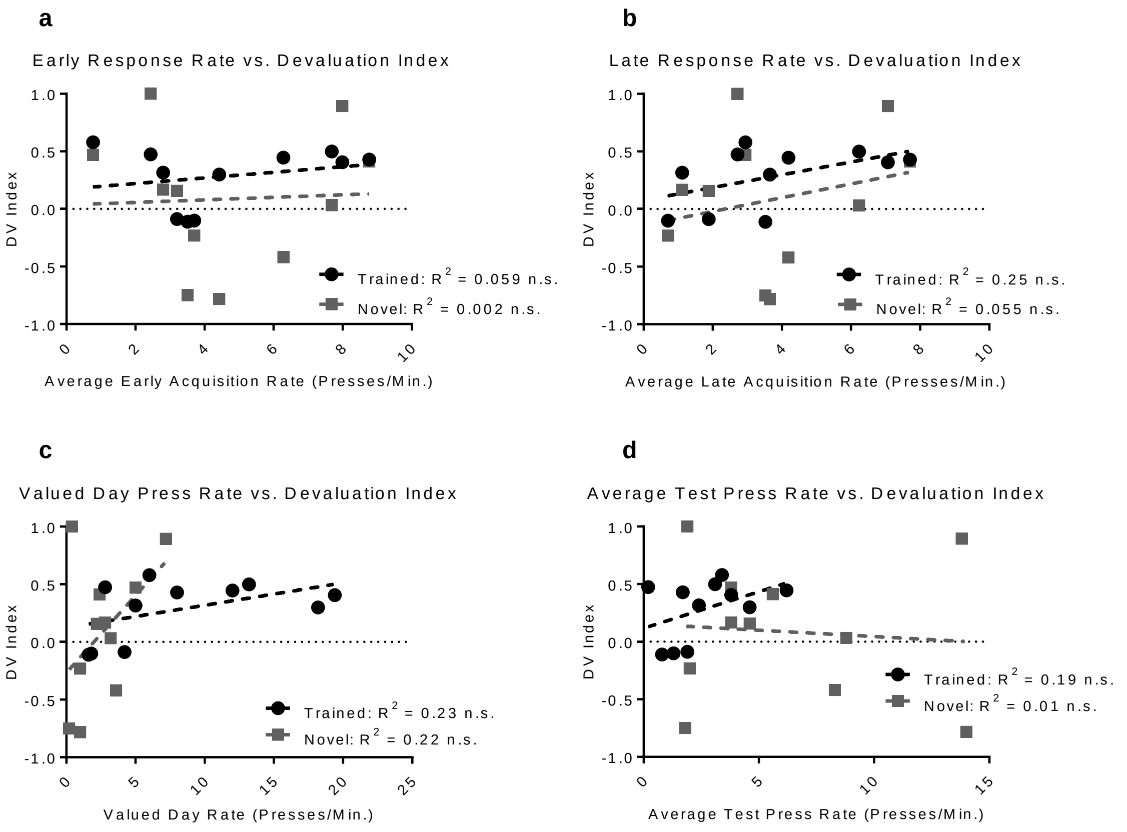

Supplementary Figure S1. No correlation between acquisition or test response rate and sensitivity to devaluation. We ran several linear regressions comparing either acquisition (**a-b**) or test (**c-d**) response rate to Devaluation Index (DV Index) to determine if response rates influenced sensitivity to devaluation for either lever. **(a)** Early acquisition response rate (average of the first two days of schedule training) vs. DV Index. Neither the trained ($F_{(1,9)} = 0.57$, p = 0.47; $R^2 = 0.059$) nor the novel ($F_{(1,9)} = 0.02$, $p = 0.89$; $R^2 = 0.002$) lever slope differed from 0. **(b)** Late acquisition response rate (final three day average) vs. DV Index. Neither the trained ($F_{(1,9)} = 2.96$, $p = 0.12$; $R^2 = 0.25$) nor the novel ($F_{(1,9)} = 0.52$, $p = 0.49$; $R^2 = 0.055$) lever slope differed from 0. **(c)** Response rate during the Valued test day vs. DV Index for the respective lever. We found no correlation between press rate on the Valued day and DV index for either the

trained ($F_{(1,9)}$ = 2.68, p = 0.14; R^2 = 0.229) or novel lever ($F_{(1,9)}$ = 2.57, p = 0.14; R^2 = 0.222). **(d)** Average response rate during test (Valued and Devalued days) vs. DV Index for the respective lever. We found no correlation between the average press rate across Valued and Devalued days and DV index for either the trained ($F_{(1,9)}$ = 2.06, p = 0.19; R^2 = 0.186) or novel lever ($F_{(1,9)}$ = 0.06, p = 0.81; R^2 = 0.007). Dotted linear regression lines indicate non-significant slopes (compared to 0). n.s. = Not Significant.

Supplementary Figure S2

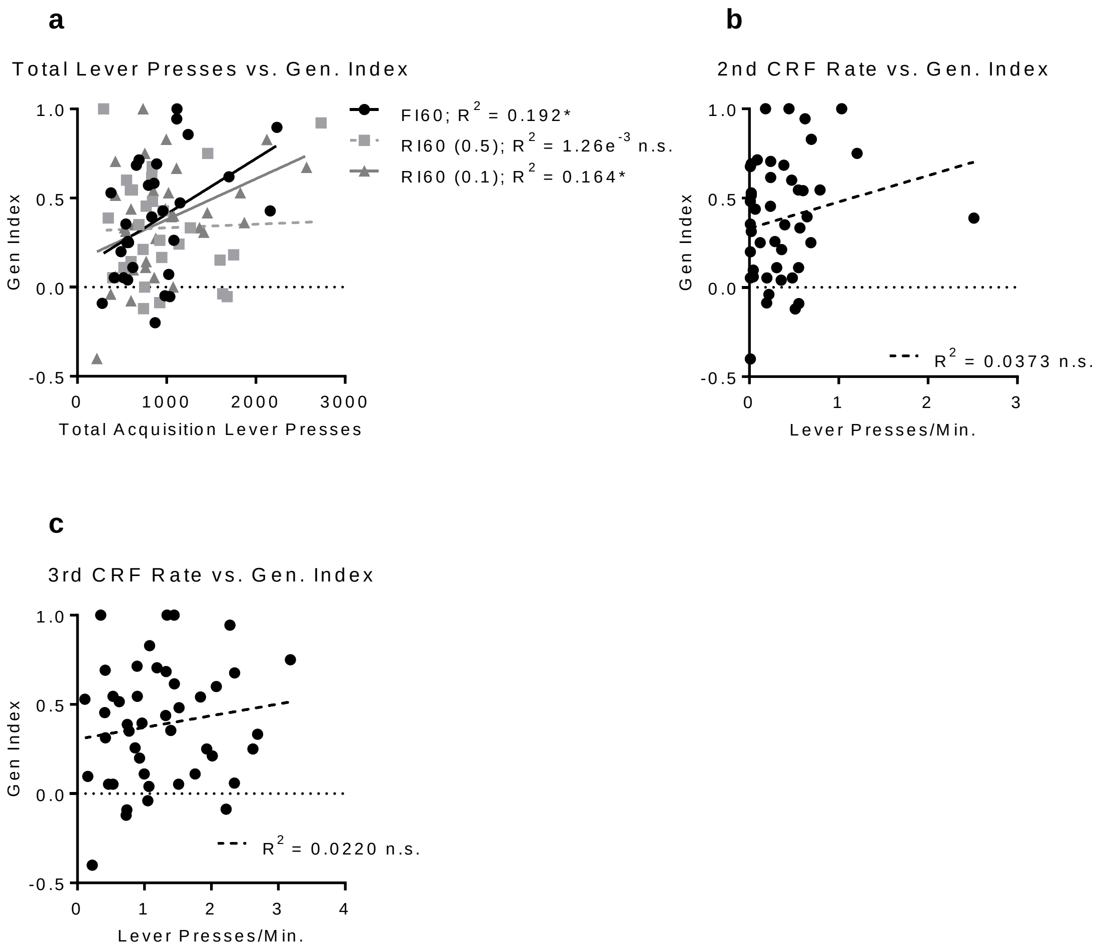

Supplementary Figure S2. Correlation between experience and exploitation exists across different schedules and is not present prior to schedule training. (a) Correlation between total lever presses during acquisition and generalization index (Gen. Index) separated by schedule. FI60 = Fixed Interval 60s. RI60 (0.5) is a Random Interval 60s schedule with moderate uncertainty. RI60 (0.1) is a Random Interval 60s schedule with high uncertainty. **(b)** Correlation between rate on the second day of CRF (Continuous Ratio of Reinforcement) training and generalization index. **(c)** Correlation between rate on the third day of CRF training and

generalization index. Dotted linear regression lines indicate non-significant slopes (compared to 0), while solid lines indicate significant slopes. n.s. = Not Significant, * = $p < 0.05$.

Supplementary Figure S3

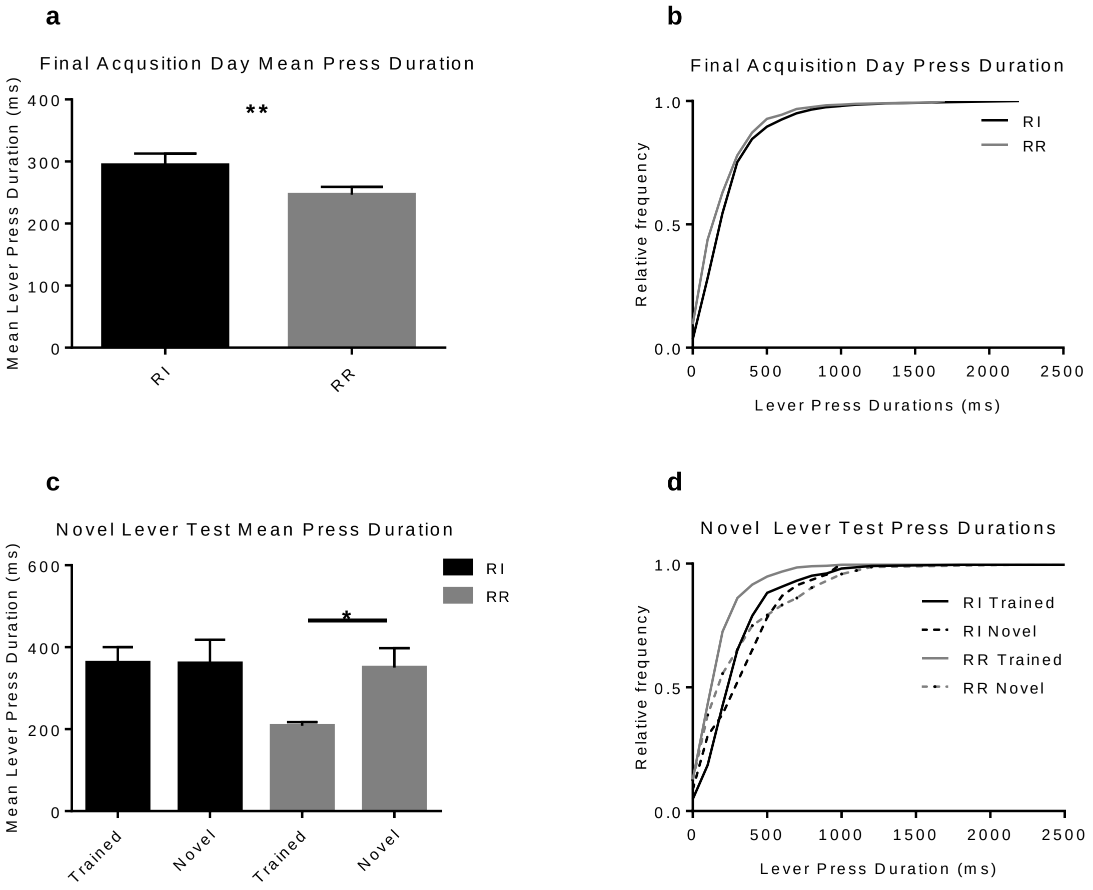

Supplementary Figure S3. Lever press duration differs between trained and novel levers in ratio-trained mice. To examine how similar action performance itself (the response) is on the trained versus novel lever, mice were trained on either a Random Ratio (RR) or Random Interval (RI) schedule and the duration of their lever presses was recorded. **(a)** Mean lever press duration on the final day of acquisition. A Mann-Whitney test of lever durations on the final day of training prior to testing revealed significant differences between the two schedules (Mann-Whitney U = 29523, n_{RI}= 202; n_{RR}= 305, p = 0.002). **(b)** Distribution of lever press durations on the final day of acquisition. **(c)** Mean lever press durations on the trained and novel lever during

the novel lever test. While mice trained under a RI schedule made trained and novel lever presses of similar durations (Mann-Whitney U = 29523, $n_{Trained} = 204$; $n_{Novel} = 23$, $p = 0.64$), those trained under a RR schedule pressed the trained and novel levers with different durations (Mann-Whitney U = 13782, $n_{Trained} = 462$; $n_{Novel} = 72$, $p = 0.02$) **(d)** Distribution of lever press durations during the novel lever test. Bars = ± SEM. n.s. = not significant, * = $p < 0.05$, ** = $p < 0.01$.
